# Therapeutic effects of antibodies to tumor necrosis factor-α, interleukin-6 and cytotoxic T-lymphocyte antigen 4 immunoglobulin in mice with glucose-6-phosphate isomerase induced arthritis

**DOI:** 10.1186/ar2437

**Published:** 2008-06-05

**Authors:** Isao Matsumoto, Hua Zhang, Takanori Yasukochi, Keiichi Iwanami, Yoko Tanaka, Asuka Inoue, Daisuke Goto, Satoshi Ito, Akito Tsutsumi, Takayuki Sumida

**Affiliations:** 1 Division of Clinical Immunology, Major of Advanced Biomedical Applications, Graduate School of Comprehensive Human Sciences, University of Tsukuba, Tennodai, Tsukuba 305-8575, Japan; 2 PRESTO, Japan Science and Technology Agency, 4-1-8 Honcho Kawaguchi, Saitama 332-0012, Japan

## Abstract

**Introduction:**

Immunization with glucose-6-phosphate isomerase (GPI) induces severe arthritis in DBA/1 mice. The present study was designed to identify the cytokines and co-stimulatory molecules involved in the development of GPI-induced arthritis.

**Methods:**

Arthritis was induced in DBA/1 mice with 300 μg human recombinant GPI. CD4^+ ^T cells and antigen-presenting cells from splenocytes of arthritic mice were cultured in the presence of GPI. Tumor necrosis factor (TNF)-α, IFN-γ, IL-2, IL-4, IL-5, IL-6, IL-10, and IL-12 levels were assessed using cytometric bead array. Monoclonal antibodies to TNF-α, IFN-γ, IL-12, CD40L, inducible co-stimulator (ICOS), and cytotoxic T-lymphocyte antigen 4 immunoglobulin (CTLA-4Ig) were used to block TNF-α and IFN-γ production, examine clinical index in mice with GPI-induced arthritis, and determine anti-GPI antibody production.

**Results:**

Large amounts of TNF-α and IFN-γ and small amounts of IL-2 and IL-6 were produced by splenocytes from mice with GPI-induced arthritis. Anti-TNF-α mAbs and CTLA-4Ig suppressed TNF-α production, whereas anti-IFN-γ mAbs, anti-IL-12 mAbs, and CTLA-4 Ig inhibited IFN-γ production. A single injection of anti-TNF-α and anti-IL-6 mAbs and two injections of CTLA-4Ig reduced the severity of arthritis in mice, whereas injections of anti-IFN-γ and anti-IL-12 mAbs tended to exacerbate arthritis. Therapeutic efficacy tended to correlate with reduction in anti-GPI antibodies.

**Conclusion:**

TNF-α and IL-6 play an important role in GPI-induced arthritis, whereas IFN-γ appears to function as a regulator of arthritis. Because the therapeutic effects of the tested molecules used in this study are similar to those in patients with rheumatoid arthritis, GPI-induced arthritis appears to be a suitable tool with which to examine the effect of various therapies on rheumatoid arthritis.

## Introduction

Rheumatoid arthritis (RA) is a chronic inflammatory disorder with variable disease outcome, and is characterized by a polyarticular inflammatory process of unknown etiology. The prognosis for RA patients has improved significantly in recent years following the introduction of tumor necrosis factor (TNF)-α antagonists [1]. Despite the increased popularity of this form of therapy, its precise mechanism of action in RA remains unclear.

Collagen-induced arthritis (CIA) is widely used as an experimental model to evaluate the effects of therapeutic agents on human RA. The effects of various anti-cytokine mAbs have been examined in this model, especially after the onset of clinical arthritis. Previous studies reported that anti-IL-1 and anti-IL-12 mAbs significantly suppressed arthritis, whereas anti-TNF-α therapy had little effect in this model [2-5], and blockade of IL-6 had no effect in established CIA [6], indicating different therapeutic mechanisms in RA [7,8].

The ubiquitously expressed self-antigen glucose-6-phosphate isomerase (GPI) was identified as an arthritogenic target in the K/B × N T-cell receptor transgenic mouse model [9,10]. Recently, immunization with human GPI was reported to provoke acute, severe arthritis in DBA/1 mice (GPI-induced arthritis), supporting the notion that T-cell and B-cell responses to GPI play a crucial role in the development of arthritis [11,12]. We recently described the presence of GPI-reactive T cells in HLA-DRB1*0405/*0901-positive patients with RA who harbored anti-GPI antibodies, a finding that emphasizes the pathogenic role of antigen-specific T cells in anti-GPI antibody-positive patients [13].

The aim of the present study was to determine the mechanism of antigen-specific arthritis. For this purpose, we analyzed the role of several cytokines and co-stimulatory molecules in GPI-induced arthritis after clinical onset. The production of TNF-α by cultured splenocytes was increased, and anti-TNF-α mAb and cytotoxic T-lymphocyte antigen 4 immunoglobulin (CTLA-4Ig) efficiently suppressed TNF-α production by splenocytes. Furthermore, a single injection of anti-TNF-α mAb and two injections (on days 8 and 12, or days 12 and 16) of CTLA-4Ig markedly reduced the severity of the disease. In contrast, neither anti-IFN-γ nor anti-IL-12 mAb altered the course of the disease. Surprisingly, a single injection of anti-IL-6 mAb resulted in cure of arthritis. Further analyses showed the presence of high serum TNF-α and IL-6 levels, but not IFN-γ and IL-1β, in arthritic mice. Moreover, effective treatment with these agents tended to reduce anti-GPI antibody production. These findings suggest that TNF-α and IL-6 play important roles in acute-onset arthritis in GPI-immunized mice. These results point to the potential roles played by these cytokines in the pathogenicity of human RA, and suggest that therapeutic strategies directed against TNF-α and IL-6 might be fruitful in RA.

## Materials and methods

### GPI-induced arthritis in DBA/1 mice

Male DBA/1 mice (aged 6 to 8 weeks) were obtained from Charles River (Yokohama, Japan). Recombinant human GPI was prepared as described previously [14]. Mice were immunized by intradermal injection of 300 μg recombinant human GPI-glutathione *S*-transferase (GST) fusion protein (hGPI) in emulsified complete Freund's adjuvant (Difco, Detroit, MI, USA). Control mice were immunized with 300 μg GST in complete Freund's adjuvant. The experimental protocol was approved by the Ethics Review Committee for Animal Experimentation of Tsukuba University School of Medicine. Arthritic animals were clinically assessed and ankle thickness recorded. We used the following arthritis scoring system to evaluate the disease state (clinical index): 0 = no evidence of inflammation, 1 = subtle inflammation or localized edema, 2 = easily identified swelling but localized to either dorsal or ventral surface of paws, and score 3 = swelling on all aspects of paws. All four limbs were evaluated using a constant tension caliper and graded, yielding a maximum possible score of 12 per mouse.

### Histological assessment of arthritis

At the indicated time points, the ankles of the mice were removed, fixed, decalcified and paraffin-embedded. Sections (5 μm) were stained with hematoxylin and eosin, and evaluated for histologic changes indicating inflammation, pannus formation, and cartilage and bone damage.

### Preparation of splenocytes and cytometric bead array

Spleens were dissected from immunized DBA/1 or B6 mice (on day 8 after immunization) and immediately immersed in phosphate-buffered saline (PBS; Gibco, Grand Island, NY, USA). Single-cell suspensions were prepared. Red blood cells were lysed by incubation of the suspension in NH_4_Cl (0.83% in 0.01 mol/l Tris-HCl [pH 7.2]). The number of splenocytes was then counted, centrifuged again, and resuspended in RPMI (Gibco, Grand Island, NY, USA). For culture, we used RPMI supplemented with 100 μg/ml streptomycin, 100 U/ml penicillin, 10% fetal bovine serum, and 50 μM 2-mercaptoethanol. After counting the cells, the medium was added to make the final concentration 2.5 × 10^6^/ml. Next, CD4^+ ^T cells were isolated by positive selection with anti-mouse CD4^+ ^antibody (T cell isolation kit; Miltenyi Biotec, Bergisch Gladbach, Germany). The labeled cells were then passed through separation columns (MiniMACS columns; Miltenyi Biotec). The cells contained more than 97% CD4^+ ^T cells. T-depleted spleen cells were treated with 50 μg/ml mitomycin C (Kyowa Hakko Kogyo, Tokyo, Japan) for 30 minutes at 37°C and were used as antigen-presenting cells (APCs).

CD4^+ ^T cells (1 × 10^6 ^cells/ml) were stimulated with 5 μg/ml GPI (or GST) and APCs (2 × 10^5 ^cells/ml) in 1 ml volume in 48-well culture plates (Nunc) for 12 hours. The culture supernatants were collected and cell-free samples were stored at -30°C until the cytokine assay. The concentrations of TNF-α, IFN-γ, IL-2, IL-4, IL-5, IL-6, IL-10, and IL-12p70 were measured using cytometric bead array (CBA) with a series of anti-cytokine mAb-coated beads and PE-conjugated anti-cytokine mAbs, followed by Epics XL flow cytometric analysis (Beckman-Coulter Electronics, Fullerton, CA, USA), using the CBA kit (BD Bioscience, San Jose, CA, USA) and software (BD).

### Antibodies used for *in vitro *and *in vivo *studies

We used commercially available anti-TNF-α mAb (eBioscience, San Diego, CA, USA; 10 μg/ml), anti-IFN-γ mAb (BD Biosciences; 1 μg/ml), and anti-IL-12 mAb (BD; 0.3 μg/ml) to neutralize the respective cytokines. These concentrations were selected based on more than 80% blockade of the respective cytokine. CTLA-4Ig (BD; 1 μg/ml), anti-inducible co-stimulator (ICOS) mAb (BD; 0.5 μg/ml), and anti-CD40L mAb (BD; 1 μg/ml) were used to block co-stimulatory pathways. As a control antibody, we used the same amount of rat IgG_1 _isotype control (R&D Systems, Minneapolis, MN, USA). Inhibition study was conducted by adding the above concentration at the start of culture. Three independent experiments were performed.

On day 8 after the onset of arthritis, each mouse received a single injection of 100 μg of anti-TNF-α mAb, anti-IL-12 mAb, anti-IFN-γ mAb or anti-IL-6 mAb. A single injection of anti-IL-6 mAb on day 14 was also administered. On the other hand, two injections of 100 μg CTLA-4Ig were administered on days 8 and 12, or on days 12 and 16 after the onset of arthritis.

### Measurement of serum levels of cytokines and anti-GPI antibodies

Serum samples were collected at the indicated time points. The serum levels of TNF-α, IL-6, IL-1β and IFN-γ were determined with the respective enzyme-linked immunosorbent assay kits (BD). To detect the levels of anti-GPI antibodies, we used hGPI and GST at 5 μg/ml (diluted in PBS) to coat microtiter plates (12 hours, 4°C). After washing twice with washing buffer (0.05% Tween 20 in PBS), Block Ace (diluted 1/4 in 1 × PBS; Dainippon Pharmaceuticals, Osaka, Japan) was used for saturation (2 hours at room temperature). After two washes, sera (diluted 1/500) were added and the plates incubated for 2 hours at room temperature. After washing, alkaline phosphatase (AP)-conjugated anti-mouse IgG (Fc-fragment specific; Jackson Immunoresearch Laboratories, West Grove, PA, USA) was added to the plate (dilution 1/5,000, 1 hour, room temperature). After three washes, color was developed with AP reaction solution (containing 9.6% diethanolamine and 0.25 mmol/l MgCl_2 _[pH 9.8]) with AP substrate tablets (Sigma Chemical Co., St. Louis, MO, USA; one AP tablet per 5 ml AP reaction solution). Plates were incubated for 30 minutes at room temperature and the optical density was measured by plate spectrophotometry at 405 nm. Determinations were conducted in triplicate, and standardized between experiments by reference to a highly positive mouse anti-GPI serum. The primary reading was processed by subtracting optical density readings of control wells (coated with GST for hGPI).

### Statistical analysis

All data were expressed as mean ± standard error of the mean. Differences between groups were examined for statistical significance by using Mann-Whitney's U test. *P *< 0.05 denoted the presence of a statistically significant difference.

## Results

### Induction of arthritis in mice immunized by recombinant human GPI

To investigate whether our own GPI immunization procedure can induce arthritis, we immunized DBA/1 mice using human recombinant GPI prepared in our laboratories. As reported previously [9,10,15], all mice developed arthritis after immunization with 300 μg recombinant GPI. Arthritis appeared at day 8, and severe arthritis was noted at day 14, with maximum ankle swelling on day 14 (data not shown). GST immunization did not induce apparent arthritis (data not shown).

### GPI induces production of TNF-α and IFN-γ by spleen cells at onset of arthritis

**Figure 1:**
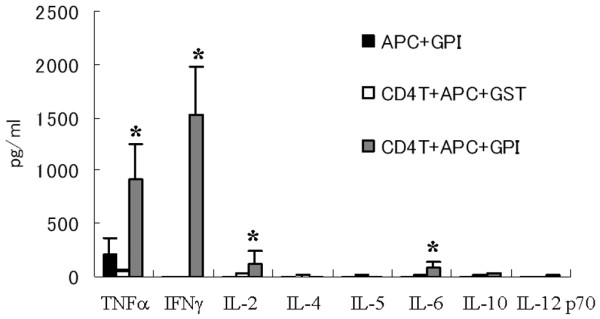
GPI-induced TNF-α and IFN-γ production from arthritic splenocytes *in vitro*. Spleens were removed from glucose-6-phosphate isomerase (GPI)-immunized DBA/1 mice (on day 8 after immunization), and then single-cell suspensions were prepared. MACS separated CD4^+ ^T cells (1 × 10^6 ^cells/ml) were stimulated with 5 μg/ml GPI (or glutathione *S*-transferase [GST]) and antigen-presenting cells (APCs; 2 × 10^5 ^cells/ml, mitomycin treated) for 12 hours. The culture supernatants were collected and concentrations of tumor necrosis factor (TNF)-α, IFN-γ, IL-2, IL-4, IL-5, IL-6, IL-10, and IL-12p70 were measured by cytometric bead array. Data were averages of three independent experiments. Error bars represent ± standard error. *P < 0.05, by Mann-Whitney U-test.

To identify the dominant cytokines at the onset of antigen-induced arthritis (day 8), we established the CBA array system using spleen CD4^+ ^T cells plus mitomycin-treated APCs cultured in GPI. In this system, treatment of APCs with mitomycin is designed to kill autoreactive APCs. The results demonstrated the production of large amounts of TNF-α and IFN-γ by the spleen of arthritic mice (Figure 1). In contrast, cells cultured with control antigen (GST) instead of GPI did not produce these cytokines (Figure 1). APC plus antigen alone produced such amounts of cytokines. Very low but detectable levels of IL-2 and IL-6 were produced, but almost no T-helper-2 type cytokines (such as IL-4, IL-5, and IL-10) were detected (Figure 1). These results indicate that exposure to the GPI antigen results in induction of TNF-α and IFN-γ by immunocytes, and suggest that these cytokines could play a crucial role in the induction of arthritis in GPI-induced mice.

### Anti-cytokine mAbs and co-stimulator blockade inhibit *in vitro *cytokine production

**Figure 2:**
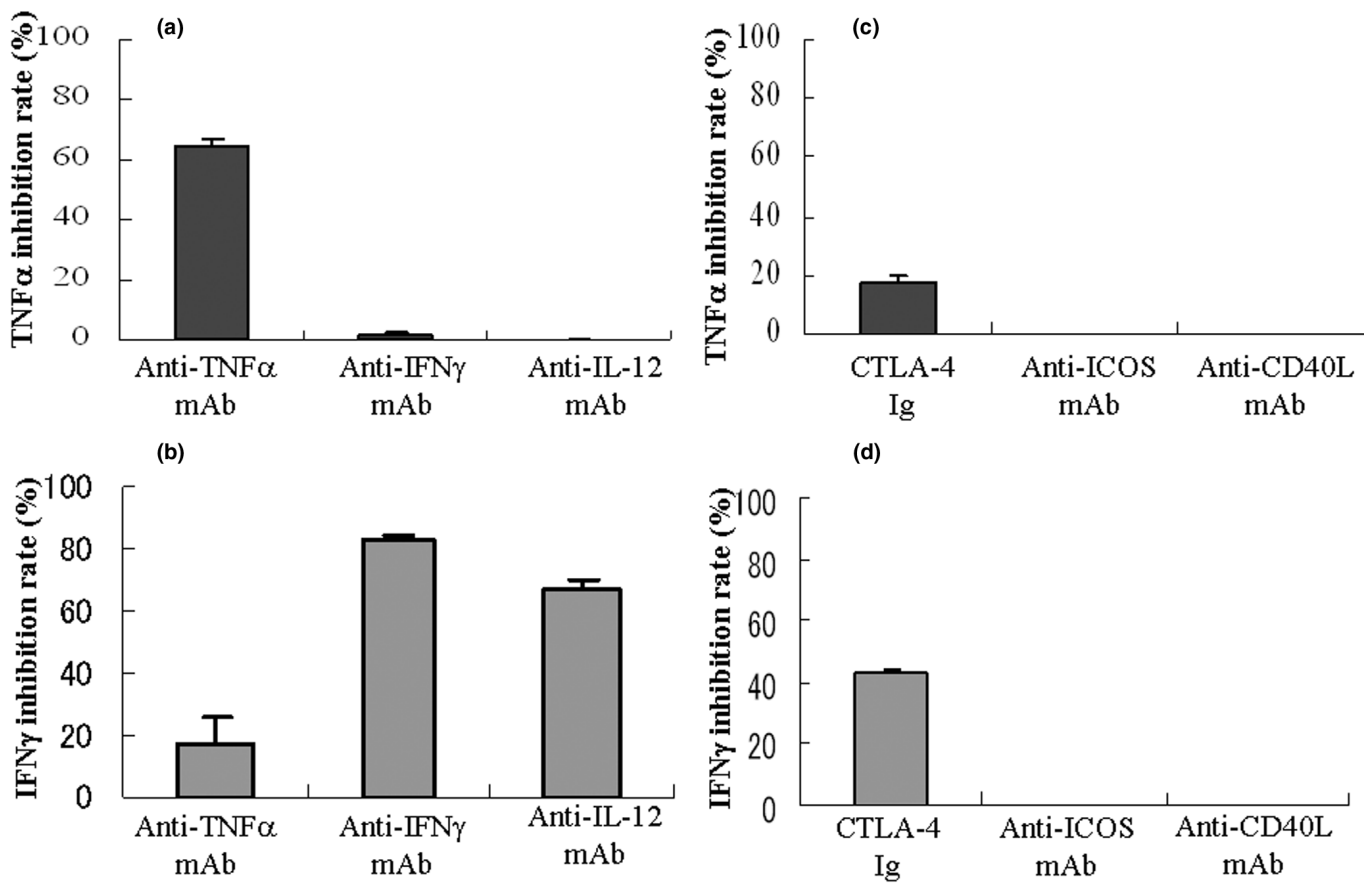
*In vitro *inhibition assay of GPI-induced TNF-α and IFN-γ production using anti-cytokine mAbs or anti-co-stimulators. High amounts of tumor necrosis factor (TNF)-α and IFN-γ were produced by splenocytes cultured with glucose-6-phosphate isomerase (GPI). Thus, we used anti-TNF-α mAb (10 μg/ml), anti-IFN-γ mAb (1 μg/ml), and anti-IL-12 mAb (0.3 μg/ml) to neutralize these cytokines in the *in vitro *cytometric bead array system. Inhibition study was conducted by adding the above concentrations at commencement of culture. These concentrations were calculated to produce more than 80% blockade of these cytokines. The percentage inhibition rate is calculated by cytokine production with this system: 100 – ([cytokine mAb – control antibody]/control antibody). The inhibition rate of **(a) **TNFα and **(b) **IFN-γ are shown. Cytotoxic T-lymphocyte antigen 4 immunoglobulin (CTLA-4Ig; 1 μg/ml), anti-inducible co-stimulator (ICOS) mAb (0.5 μg/ml), and anti-CD40L mAb (1 μg/ml) were also used to block co-stimulatory pathways, and the inhibition rate of **(c) **TNF-α and **(d) **IFN-γ are shown. Three independent experiments were performed. Data are expressed as mean ± standard error of the mean.

To delineate the separate contributions of TNF-α and IFN-γ, we performed blocking experiments using neutralizing mAbs for anti-TNF-α, IFN-γ, and IL-12 using the CBA array system. TNF-α production was inhibited by anti-TNF mAb (64.7 ± 2.7%; Figure 2a), but not by anti-IL-12 mAb (0%; Figure 2a). On the other hand, IFN-γ production was inhibited by anti-IFN-γ mAb (82.5 ± 1.2%; Figure 2b) as well as by anti IL-12 mAb (67.5 ± 2.5%; Figure 2b), and weakly by anti-TNF-α mAb (17.2 ± 9.2%; Figure 2b). These results suggest that TNF-α production is not regulated by IFN-γ, although IFN-γ is partially regulated by TNF-α.

To determine the effect of co-stimulatory molecules in established arthritis, we conducted the same *in vitro *experiments by using CTLA-4Ig, anti-ICOS, and anti-CD40L mAbs. CTLA-4Ig suppressed TNF-α (18 ± 2.1%; Figure 2c), and IFN-γ (42.9 ± 2.1%; Figure 2d) production, but not anti-ICOS or anti-CD40L mAb. These findings suggest that the antigen-induced cytokines are mainly driven by CD28/B7-1,2 co-stimulator.

### Treatment of GPI-induced arthritis with anti-TNF-α mAb

**Figure 3:**
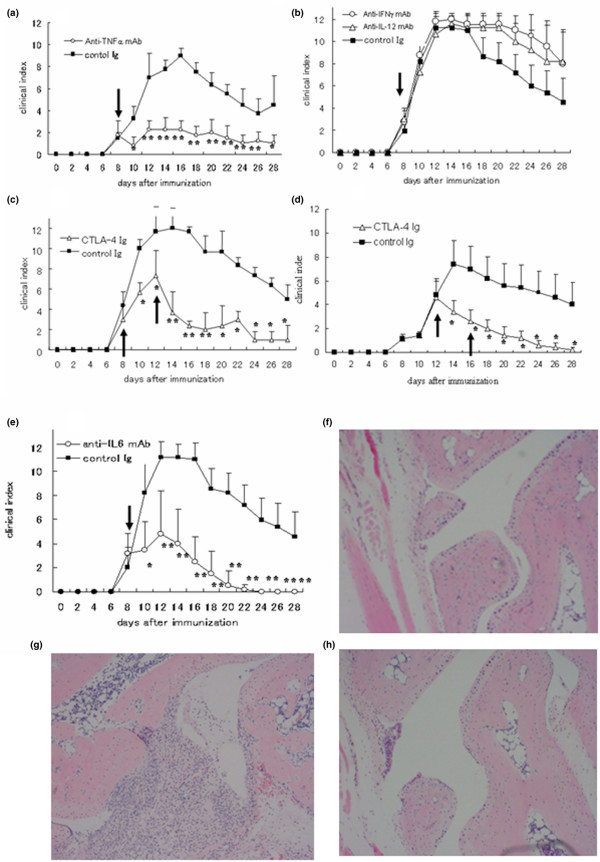
Therapeutic effect of anti-TNF mAb, CTLA-4Ig, and anti-IL-6 mAb in GPI-induced arthritis. Glucose-6-phosphate isomerase (GPI)-immunized mice were treated with **(a) **anti-tumor necrosis factor (TNF)-α mAb; **(b) **anti-IFN-γ mAb or anti-IL-12 mAb; **(c) **cytotoxic T-lymphocyte antigen 4 immunoglobulin (CTLA-4Ig; on days 8 and 12); **(d) **CTLA-4Ig (on days 12 and 16); and **(e) **or anti-IL-6 mAb just after the onset of arthritis (on day 8, on days 8 and 12, or days 12 and 16; arrow). The mean clinical index (± standard error) was examined throughout the study. *P < 0.05, **P < 0.01, by Mann-Whitney's U test. n = 6 mice in each group. Hematoxylin and eosin staining at day 21 (×40) is also shown: **(f) **anti-TNF-α mAb, **(g) **control antibody, and **(h) **CTLA-4Ig (on days 8 and 12).

To identify the pathogenic cytokine that can provoke the onset of arthritis, we conducted *in vivo *experiments using neutralizing mAbs. A single injection of 100 μg of anti-TNF-α mAb at day 8 ameliorated the disease (Figure 3a). In contrast, injection of the same dose of anti-IFN-γ or anti-IL-12 mAb had no such effect on the course of the disease, but rather tended to exacerbate the arthritis (Figure 3b). Histopathological examination of the joints of treated mice showed a clear therapeutic effect for anti-TNF-α mAb (Figure 3f, on day 21) as compared with that of control antibody (Figure 3g, on day 21). These results suggest that TNF-α blockade has clear therapeutic effect in GPI-induced model, irrespective of the minor role of 'conventional' T-helper-1 autoimmunity.

### Treatment of GPI-induced arthritis with CTLA-4Ig and anti-IL-6 mAb

**Figure 4:**
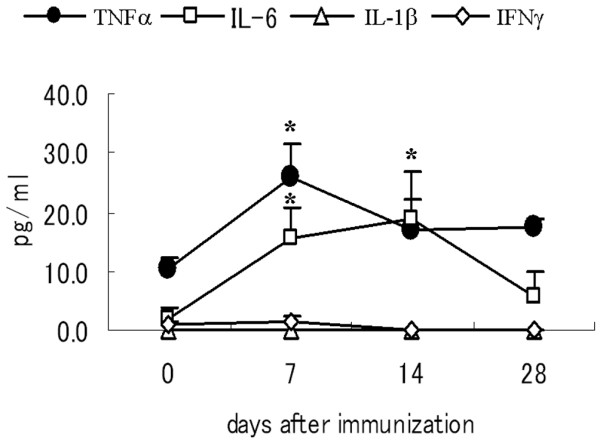
Concentration of inflammatory cytokines in serum of mice with GPI-induced arthritis. After immunization with glucose-6-phosphate isomerase (GPI), serum samples were collected from GPI-induced DBA/1 mice at the indicated time points (days 0, 7, 14, and 28). Serum concentrations of tumor necrosis factor (TNF)-α (solid circle), IL-6 (open square), IL-1β (open triangle), or IFN-γ (open diamond) were determined by enzyme-linked immunosorbent assay. Data are expressed as mean ± standard error. *n* = 3 mice in each group. *P < 0.05, by Mann-Whitney's U-test.

To investigate the effect of CTLA-4Ig *in vivo*, we treated arthritic mice with CTLA-4Ig on days 8 and 12, or on days 12 and 14. A marked improvement was seen after the second treatment (on days 8 and 12), probably because of a reduction in effector T cells at that stage (Figure 4c, and hematoxylin and eosin staining on day 21 in Figure 4h). Moreover, if we admininstered treatment on days 12 and 16, clear therapeutic efficacy was observed after the first treatment. This finding suggests that CTLA-4Ig is also therapeutically potent, especially on day 12, in mice with GPI-induced arthritis.

IL-6 is also an important cytokine in arthritis, and it is considered a promising target for the treatment of RA [7,8]. Serum IL-6 concentrations were elevated in arthritic mice, especially during the disease effector phase (Figure 4). In the next step, we assessed the effect of IL-6 blockade in mice with GPI-induced arthritis. Surprisingly, anti-IL-6 treatment on day 8 resulted in improvement in the clinical index (Figure 3e), although treatment on day 14 had no effect on the course of the disease (data not shown), suggesting that IL-6 is also pathologically crucial in the early effector phase in arthritis.

### Role of various inflammatory cytokines in GPI-induced arthritis

To determine the effects of inflammatory cytokines during the effector phase of arthritis, we measured the serum concentrations of TNF-α, IL-6, IL-1β, and IFN-γ at days 0, 7, 14, and 28 in DBA/1 mice after GPI immunization. Serum TNF-α concentration was upregulated at disease onset (day 7), but gradually decreased to the basal level by day 28 (Figure 4). On the other hand, serum IL-6 concentration was upregulated gradually, especially during the disease effector phase (days 7 and 14; Figure 4). In contrast, serum IL-1β and IFN-γ concentrations were persistently low and below the detection limit (4 pg/ml) in GPI-induced mice throughout the study (Figure 4). These findings suggest a systemic TNF-α/IL-6 imbalance in arthritic mice.

### Effective treatments tend to alter anti-GPI antibody production

**Figure 5:**
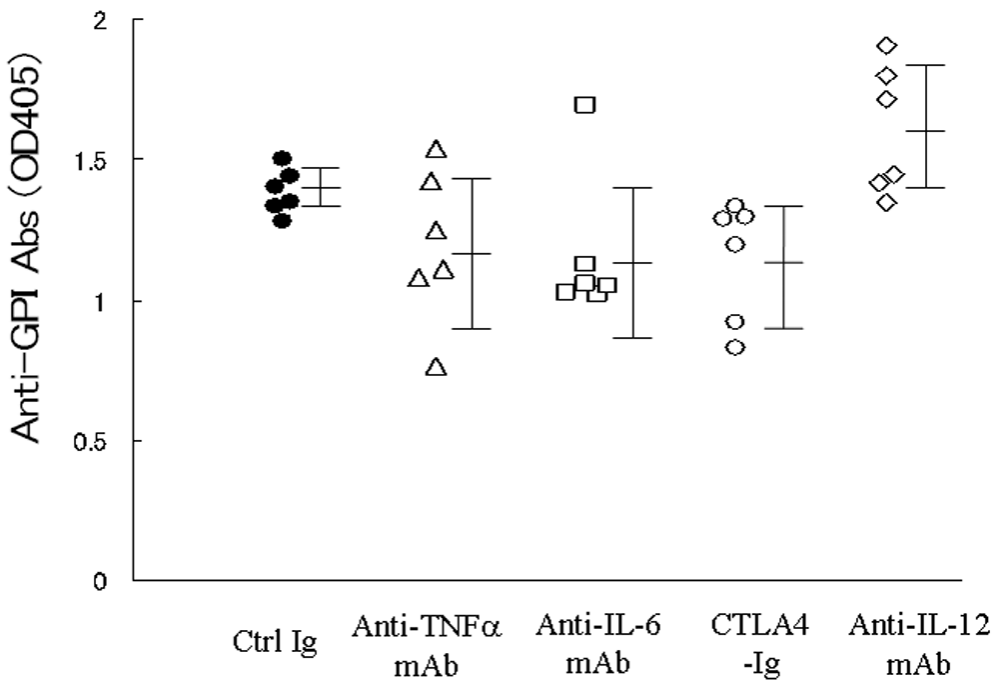
Effective treatments tend to alter anti-GPI antibody production. Glucose-6-phosphate isomerase (GPI)-induced arthritic mice were treated with 100 μg anti-tumor necrosis factor (TNF)-α mAb, anti-IL-6 mAb, cytotoxic T-lymphocyte antigen 4 immunoglobulin (CTLA-4Ig), and anti-IL-12 mAb on day 8, and CTLA-4 Ig on day 12. Serum samples were collected on day 14. The titers of anti-GPI antibodies were analyzed by enzyme-linked immunosorbent assay. Each symbol represents a single animal. Data are expressed as mean ± standard deviation of optical density.

Anti-GPI antibodies have potent arthritogenic capacity in K/B × N mice. However, anti-GPI antibodies from mice with GPI-induced arthritis do not solely cause arthritis (Schubert and coworkers [11] and our preliminary observations). In GPI-induced arthritis, IgG and C3 are co-localized on the articular surface of arthritic joints (Tanaka and coworkers, unpublished data). Accordingly, we compared the effects of anti-cytokine mAbs, immunomodulatory molecule CTLA-4Ig, and control immunoglobulin on the production of anti-GPI antibodies in mice with GPI-induced arthritis. The antigen was injected on day 8, and then sera were collected on day 14. As shown in Figure 5, anti-TNF-α, anti-IL-6, and CTLA-4Ig tended to suppress the production of anti-GPI antibodies, whereas IL-12 mAb slightly enhanced the production of the antibodies. These findings suggest that effective treatments might also alter autoantibody production during this phase of GPI-induced arthritis.

## Discussion

GPI, a ubiquitous glycolytic enzyme, is a new candidate autoantigen in inflammatory arthritis, initially identified in K/B × N mice [10]. In K/B × N mice, anti-GPI antibodies solely induce arthritis through activation of complements and Fcγ receptors [16]. With regard to cytokine dependency, anti-TNF-α mAb does not prevent the development of arthritis in K/B × N mice, and IL-6 deficiency has no influence on the development of arthritis by K/B × N serum transfer [17]. Based on this cytokine dependency, K/B × N mice differ from patients with RA.

Although the therapeutic effect of TNF antagonists has been established in RA, there are few animal models of arthritis in which TNF antagonists are confirmed as being therapeutically beneficial. For example, in the most conventional RA models, such as CIA, treatment with IL-1 antagonists significantly suppressed arthritis, whereas TNF antagonists had minor effect [2-4]. On the other hand, a clear therapeutic effect of anti-TNF-α mAb was reported recently in DNaseII-type I IFN double knockout mice [18], although this was not a genetically unaltered mouse. Schubert and coworkers [11] reported that continuous injections of human TNF receptor p75-IgG-Fc fusion protein (etanercept) from days 0 to 9 completely protected against the development of arthritis in GPI-induced arthritis. In this regard, we demonstrated a clear therapeutic effect for TNF antagonist in mice with GPI-induced arthritis, and the therapeutic response correlated with the *in vitro *regulation of TNF production. For example, we detected specific TNF-α-induced molecules in spleen and joints of mice with GPI-induced arthritis by Genechip analysis (Matsumoto and Inoue, unpublished data). These results also indicate that the GPI-induced arthritis model is suitable tool for studying the mechanisms of action of TNF-α antagonists in RA patients.

CTLA-4Ig can selectively modulate the CD80 or CD86-CD28 co-stimulatory signal required for full T-cell activation [19], and is a promising new molecule for treatment of RA [19-21]. Although administration of CTLA-4Ig at the time of immunization prevented the development of CIA, the therapeutic efficacy has not been clearly confirmed in this model [22]. In the present study, we demonstrated that only two injections of CTLA-4Ig (both on days 8 and 12 or on days 12 and 16) markedly prevented the development of arthritis in mice with GPI-induced arthritis. What is the mechanism of action of CTLA-4Ig in GPI-induced arthritis? We recently reported that anti-IL-17 mAb is also therapeutically promising in this model [15], and thus effector T-helper-17 dependency is much stronger than in the CIA model. The present study showed that treatment with CTLA-4Ig resulted in suppression of anti-GPI antibody production. Therefore, blockade of persistent T-cell activation during the early effector phase appears therapeutically useful in GPI-induced arthritis, through inhibition of both effector T-helper-17 cells and autoantibody production.

Like TNF-α and IL-1, IL-6 is a pleiotropic cytokine that is known to play a role in RA, and a humanized anti-IL-6 receptor antibody (tocilizumab) was recently reported to be beneficial therapeutically [7,8]. However, administration of IL-6 antagonist did not produce any remedial effects when administered after the onset of arthritis in CIA animals [6]. In the present study we demonstrated that treatment with anti-IL-6 mAb inhibited the development of arthritis and even after the onset of arthritis in mice with GPI-induced arthritis. However, anti-IL-6 mAb had no effect on day 14, even if we used 4 mg anti-IL-6 receptor mAb [15]. Our results with the *in vitro *CBA assay showed that IL-6 was not the main cytokine produced by antigen cultures. Cultures of the same numbers of mitomycin-untreated and -treated splenocytes with GPI showed that IL-6 was predominantly produced by whole spleen cells, indicating that mitomycin-sensitive APCs, including B cells, were the major source of IL-6 (data not shown). Another study showed that IL-6 antagonism on day 8 suppressed the proliferation of antigen-specific T cells and partially the development of T-helper-17 cells, with reduced production of anti-GPI antibody [15]. Therefore, the effectiveness by IL-6 antagonist on day 8 in GPI-induced arthritis appears to be mediated through orchestration of these mechanisms.

In the GPI-induced arthritis model, anti-GPI antibodies could not induce arthritis on their own. Neither Fcγ receptor deficient nor B-cell-deficient mice had overt arthritis [11,12], suggesting that anti-GPI antibodies play an indispensable role in this model. Recent studies identified co-localization of IgG and C3 on the articular surface of joints in GPI-induced arthritis on day 14, and production of anti-GPI antibodies was most vigorous on day 8 (Tanaka and coworkers, unpublished data). These results mimic those of arthritis mediated by K/B × N serum transfer [23]. Thus, we investigated whether immunomodulatory molecules could alter this vigorous antigen-specific antibody production on day 8. Treatment of mice with CTLA-4Ig resulted in downregulation of anti-GPI antibody production, whereas anti-TNF-α and anti-IL-6 mAb therapy tended to reduce these antibodies. In contrast, anti-IL-12 mAb rather upregulated the production of anti-GPI antibodies, leading to persistent arthritis. These findings suggest that production of anti-GPI antibodies in the early effector phase may correlate with the severity of arthritis in this model.

Does this model mimic human RA, especially in anti-GPI antibody-positive individuals? Severe forms of RA have been described in patients with high titers of anti-GPI antibodies, although these antibodies were also identified in a few control individuals [14,24]. In anti-GPI antibody-positive individuals, GPI-reactive CD4^+ ^T cells, especially T-helper-1 type cells, were detected among peripheral blood mononuclear cells of RA patients with either HLA-DR 0405 or 0901 haplotype [13]. What about GPI-induced arthritis? High titers of anti-GPI antibodies are present in arthritis-resistant C57BL/6 mice (H-2^b^) [11,12], although the T cells of these animals exhibited weak GPI responses compared with arthritis-susceptible DBA/1 mice (H-2^q^). These results indicate that anti-GPI antibodies cannot themselves induce arthritis; it is likely that a unique H-2 haplotype and activation of antigen-specific T cells are necessary for the development of arthritis in this model. Moreover, the effectiveness of CTLA-4Ig was clearly similar to that in human RA. Considered together, GPI-induced arthritis seems to be akin to human RA.

## Conclusion

Because the therapeutic effects of the tested biologics used in this study are similar to those in patients with RA, GPI-induced arthritis is a suitable model for examining the pathogenic mechanisms of RA and the effect of various treatments.

## Abbreviations

AP = alkaline phosphatase; APC = antigen-presenting cell; CBA = cytometric bead array; CIA = collagen-induced arthritis; CTLA-4Ig = cytotoxic T-lymphocyte antigen 4 immunoglobulin; GPI = glucose-6-phosphate isomerase; GST = glutathione S-transferase; hGPI = recombinant GPI-GST fusion; ICOS = inducible co-stimulator; IFN = interferon; IL = interleukin; mAb = monoclonal antibody; PBS = phosphate-buffered saline; RA = rheumatoid arthritis; TNF = tumor necrosis factor.

## Competing interests

The authors declare that they have no competing interests.

## Authors' contributions

IM wrote the manuscript and conceived of the study. HZ, TY, KI, YT, and AI performed all experiments and coordinated the statistical study. TH participated in clinical assessment. TS participated in its full design and coordination, and DG, SI and AT participated in discussions.
